# Development of functional markers and expression analysis for a Peroxidase gene *TaPod‐A3* on chromosome 7AL in wheat

**DOI:** 10.1002/tpg2.70103

**Published:** 2025-08-30

**Authors:** Xinyuan Liu, Zhaoqiang Wang, Lili Wang, Yukun Cheng, Bin Bai, Hongwei Geng, Mengyao Ma

**Affiliations:** ^1^ High Quality Special Wheat Crop Engineering Technology Research Center of Xinjiang Agricultural University Urumqi China; ^2^ Joint International Research Laboratory of Crop Biological Breeding along the Silk Road Economic Belt Urumqi China; ^3^ Xinjiang Key Laboratory of Crop Biological Breeding Urumqi China; ^4^ Wheat Research Institute, Gansu Academy of Agricultural Sciences Lanzhou China

## Abstract

Peroxidase (POD) is one of the key factors affecting the wheat flour quality. Characterization and development of functional markers, as well as expression analysis of POD genes, will help in breeding wheat cultivars and advanced lines with better flour quality. Here, we cloned a POD gene, *TaPod‐A3*, on chromosome 7AL and developed its functional marker in common wheat (*Triticum aestivum* L). Based on single nucleotide polymorphisms (SNPs) and Indel between *TaPod‐A3* allele sequences, functional markers *POD‐7A1*, *POD‐7A2*, and *POD‐7A3* were developed, amplifying 216, 882, and 156 bp fragments in wheat cultivars and advanced lines with lower, middle, and higher POD activities, respectively. The analysis of variance of 228 wheat cultivars and advanced lines showed that the mean POD activity (668.6 U min^−1^ g^−1^) of 113 wheat cultivars and advanced lines supplemented with *TaPod‐A3a* was lower than 17 wheat cultivars and advanced lines supplemented with *TaPod‐A3b* (679.7 U min^−1^ g^−1^) and the 98 wheat cultivars and advanced lines supplemented with *TaPod‐A3c* (731.2 U min^−1^ g^−1^). A total of 228 wheat cultivars and advanced lines were found using the functional markers of *TaPod‐A1*, *TaPod‐D1*, and *TaPod‐A3* genes located on chromosomes 3A, 7D, and 7AL of the functional markers developed in this study. The wheat cultivars and advanced lines with favorable allele combination of *TaPod‐A1b/TaPod‐A3c/TaPod‐D1b* had higher POD activity (mean POD activity 780.6 U min^−1^ g^−1^) than those with alleles *TaPod‐A1a/TaPod‐A3a/TaPod‐D1a* (625.7 U min^−1^ g^−1^). Six wheat cultivars and advanced lines with the same genotype and phenotype were selected for quantitative real‐time polymerase chain reaction, and we found that the expression level of F49‐70 in wheat cultivars and advanced lines with high POD activity was significantly higher than that in Wanmai 29 with low POD activity at each stage after flowering (*p* < 0.05). Based on correction analyses on the *TaPod‐A3* gene expression, the expression level was positively correlated with POD activity. This study provides useful information on the POD genes in bread wheat, insight into the *TaPod‐A3* gene structure and functional markers, as well as valuable resources for improving the quality of wheat flour.

## Core Ideas

- By conducting bioinformatics analysis on the *TaPod‐A3* gene, its gene structure is clarified.
- We developed single nucleotide polymorphism (SNP)/Indel‐based markers (POD‐7A1/2/3) to distinguish low (216 bp), medium (882 bp), and high (156 bp) POD activity genotypes.
- Allele *TaPod‐A3c* increased POD activity (731.2 > 668.6 U·min^−1^·g^−1^), while the optimal haplotype (*TaPod‐A1b/A3c/D1b*) synergistically boosted activity.
- *TaPod‐A3* expression during grain development correlated with POD activity (*p* < 0.05), confirming transcriptional regulation.

## Plain Language Summary

Peroxidase (POD) influences wheat flour quality. This study cloned the *TaPod‐A3* gene on chromosome 7AL and developed functional markers (*POD‐7A1*, *POD‐7A2*, *POD‐7A3*) based on single nucleotide polymorphisms (SNPs)/Indel, distinguishing wheat lines with low (216 bp), medium (882 bp), and high (156 bp) POD activity. Analysis of variance of 228 wheat lines revealed *TaPod‐A3c* had the highest mean POD activity (731.2 U·min^−1^·g^−1^), while *TaPod‐A3a* had the lowest (668.6 U·min^−1^·g^−1^). Lines with the *TaPod‐A1b/A3c/D1b* allele combination showed higher POD activity (780.6 U·min^−1^·g^−1^) than *TaPod‐A1a/A3a/D1a* (625.7 U·min^−1^·g^−1^). Quantitative real‐time polymerase chain reaction confirmed higher *TaPod‐A3* expression in high POD lines, correlating with activity. This work provides functional markers and insights for wheat quality improvement.

**Abbreviations**
POD: peroxidase
SNPs: single nucleotide polymorphisms
ANOVA: analysis of variance
QTL: quantitative trait locus
qPCR: quantitative real‐time polymerase chain reaction

## 1 INTRODUCTION

Wheat (*Triticum aestivum* L.) is an important food crop worldwide, providing staple food for the world's population (Liu et al., 2024). Multiple factors affect flour color, including yellow pigment content, oxidase activity, and protein content (Zhou et al., 2021). Class III peroxidase (POD) is one of the important factors affecting the quality of wheat flour (Zhou et al., 2021). A plant‐specific oxidoreductase is widely distributed in cereals, it is also an important cause of browning and bleaching of wheat flour and flour products (Hiraga et al., 2001; Zhai et al., 2013).

POD in wheat is distributed in multiple tissues, including roots, stems, leaves, flowers, grains, and embryos. Previous studies have shown that PODs are involved in various aspects of plant physiology, such as participating in the metabolism of reactive oxygen species, the synthesis of lignin and suberin, resistance to insect attack, resistance to stress, as well as the generation and detoxification of reactive oxygen species forms (Daudi et al., 2012; Shigeto & Tsutsumi, 2016). In addition, POD has turned out to be an effective catalyst for the water‐soluble oxidative gelatinization of pentosan in wheat flour (Crowe & Rasper, 1988; Izydorczyk et al., 1990). It catalyzes tyrosine cross‐linking to improve the ductility, viscosity, and elasticity of dough, and can be used for wheat quality improvement (Pico et al., 2015; Shu et al., 2012; Takasaki et al., 2005). Through its effect on flour, POD thus has a significant effect on the quality of noodles and related products and is highly positively correlated with the browning degree of dough and pasta products (Feillet et al., 2000; Geng et al., 2012; Hemalatha et al., 2007). Furthermore, POD can replace lipoxygenase as the main food additive due to causing less damage to the flavor of flour products (Gélinas et al., 1998; Hidalgo et al., 2010).

Common wheat is a typical heterohexaploid plant (Mujeeb‐Kazi et al., 2009; Takasaki et al., 2005). In addition, POD traits go through quantitative trait, inheritance, and are mainly influenced by genetic factors followed by environmental factors (Feillet et al., 2000; Zhou et al., 2021). Therefore, it is feasible to increase the POD activity of wheat flour through genetic improvement. The following are some findings from various earlier studies for this purpose. Several POD genes were mapped on chromosomes 2A, 2B, 2D, 4B, 7A, and 7D by using the Chinese Spring (CS)‐nullitetrasomic lines (Kobrehel & Feillet, 1975). The functional markers *POD‐7D1* and *POD‐7D6* of *TaPod‐D1* were developed to target the genes with the highest contribution rate on chromosome 7D (Geng et al., 2019). Conversely, functional markers for the major‐effect genes on chromosome 3A were developed based on *TaPod‐A1 *(Wei et al., 2015). Allelic variation analysis of 110 Xinjiang wheat varieties (lines) using functional markers for the loci of *TaPod‐A1*, *TaPod‐A3*, and *TaPod‐D1* revealed that the benefit allele combination (*TaPod‐A1b/TaPod‐A3c/TaPod‐D1b*) exhibited significantly higher POD activity (2836.25 U min^−1^ g^−1^) compared to those with the non‐benefit alleles (*TaPod‐A1a/TaPod‐A3a/TaPod‐D1a*, 2210.69 U min^−1^ g^−1^) (*p* < 0.01). Furthermore, the POD activity of the benefit multi‐locus haplotype surpassed that of any single‐locus allele, demonstrating that allelic stacking enhances POD activity (X. Y. Liu et al., 2025). In addition, the multiple reference‐quality genome assemblies, and the exploration of genomic variation, have improved the identification of new wheat genes and the development of their functional markers. Fluorescence quantitative real‐time polymerase chain reaction (qPCR) technology can exclude the influence of environment on gene expression, and it has been reported that qPCR was performed on genes with different expression patterns in different tissue wheat cultivars and advanced lines, at different periods, and with different treatments (Ferreira et al., 2023; Tang et al., 2020).

The POD genes of wheat are a large family of genes, and therefore more POD genes need to be accurately localized to manipulate POD activity and improve the quality of the final utilization of wheat. Currently, there are relatively few studies on wheat POD genes, and only a few wheat POD genes have been cloned, with functional markers developed, to control the quantitative trait locus (QTL) of POD activity. Here, we aim to (i) clone and analyze the full‐length sequence of the *TaPod‐A3* gene, (ii) establish robust functional markers for *TaPod‐A3*, and (iii) explore the correlation between *TaPod‐A3* expression level and POD activity and demonstrate its utility for marker‐assisted selection.

## 2 MATERIALS AND METHODS

### 2.1 Wheat cultivars and advanced lines

The genome of wheat cultivar CS was used to clone the full length of *TaPod‐A3*. A total of 228 wheat cultivars and advanced lines (58 foreign winter wheat cultivars and advanced lines and 170 domestic wheat cultivars and advanced lines) were used to validate the association between the allelic variation and POD activity of *TaPod‐A1*, *TaPod‐A3*, and *TaPod‐D1*. The functional markers *POD‐3A1* and *POD‐3A2* located in the *TaPod‐A1* gene and *POD‐7D1* and *POD‐7D6* located in the *TaPod‐D1* gene were developed by the members of the research group (Geng et al., 2019; Wei et al., 2015). POD activity in wheat grains was assayed following the protocols from Wei et al. (2015). The field management followed the local practices. qPCR analysis was performed on six wheat genotypes exhibiting consistent POD activity phenotypes, including cultivars Wanmai 29, Zhongyu 5, Kechengmai 1, F49‐70, and Shan 354.

### 2.2 POD activity assay

The POD activity in wheat grains was determined using guaiacol ultraviolet spectrophotometry (Wei et al., 2015). In summary, 1.2 g of decontaminated, defect‐free grains were ground into whole wheat flour, from which 0.5 g aliquots were analyzed in triplicate using a Molecular Devices Spectra Max 384 Plus spectrophotometer (LLC) at 470 nm. POD activity was defined as the Δ_470_/min/g flour required to produce a 0.01 absorbance increase, with final values representing the mean of three parallel determinations from replicate flour extracts.

**Core Ideas**

- By conducting bioinformatics analysis on the *TaPod‐A3* gene, its gene structure is clarified.
- We developed single nucleotide polymorphism (SNP)/Indel‐based markers (POD‐7A1/2/3) to distinguish low (216 bp), medium (882 bp), and high (156 bp) POD activity genotypes.
- Allele *TaPod‐A3c* increased POD activity (731.2 > 668.6 U·min^−1^·g^−1^), while the optimal haplotype (*TaPod‐A1b/A3c/D1b*) synergistically boosted activity.
- *TaPod‐A3* expression during grain development correlated with POD activity (*p* < 0.05), confirming transcriptional regulation.

### 2.3 DNA and RNA isolation

The gDNA was extracted according to the SDS‐Tris method with minor modifications (Kang et al., 1998). Total RNA was extracted from developing wheat grains at key grain‐filling stages (7, 14, 21, 28, and 35 days after flowering [DAF]) using the TransZol Up Plus RNA Kit (TransGen Biotech). The time points correspond to the following stages of grain development: early milk stage (7 DAF), late milk stage (14 DAF), soft dough stage (21 DAF), hard dough stage (28 DAF), and physiological maturity (35 DAF). These stages cover the major phases of grain development from active storage accumulation to maturation. The purity of RNA was measured by a nucleic acid meter (OD260/OD280 between 1.8 and 2.2).

### 2.4 Sequence analysis of POD gene *TaPod‐A3*

The full‐length sequences of the *TaPod‐A3* gene (TraesCS7A02G339600) on chromosome 7AL were originally searched in EnsemblPlants and was successfully cloned based on the downstream sequence on chromosome 7AL (Geng et al., 2019). The wheat genome annotation file was downloaded from EnsemblPlants (http://plants.ensembl.org/index.html) after searching for genes among wheat species similar to *TaPod‐A3* in Wheatomics 1.0. Gene structures were localized by the Gene Structure Display Server (http://gsds.gao‐lab.org/). Phylogenetic analysis was constructed using MEGA 11.0 and the Neighbor‐Jointing method was used to construct the genes building a phylogenetic tree (bootstrap, 1000).

### 2.5 Allelic of the *TaPod‐A3* gene in wheat

Four wheat cultivars and advanced lines with a broad range of POD activities, Chinese spring/Zhong 892, Doumai/Shi 4185, Zhoumai 16/Gaocheng 8901, and Linmai 2/Zhou 8425B, were used for the development of sequence tagged sites markers. Based on the polymorphism between sequences, we developed the markers *POD‐7A1*, *POD‐7A2* and *POD‐7A3* to identify the variants. The chromosome‐specific primers were designed using Primer 5.0 and synthesized by Shanghai Sheng gong Biotechnology Co., Ltd. (http://www.sangon.com/). The PCR products were cloned into PEASY‐T5 vectors under the following thermal cycling conditions: 94°C for 5 min, followed by 35 cycles at 94°C for 30 s, 65°C–68°C for 45 s, 72°C for 1 min, and finally 72°C for 8 min. The PCR mix comprised of 1 µL of gDNA, 1 µL of forward primer (µmol·L^−1^), 1 µL of reverse primer (µmol·L^−1^), 12.5 µL of 2×Taq‐Master Mix, 9.5 µL of water, with total volume of 25 µL.

### 2.6 RT‐qPCR analysis

RT‐PCR was utilized to detect the expression of POD genes in six wheat cultivars and advanced lines (namely, the three cultivars Soissons, Kechengmai1, and F49‐70 with high POD activity, and the three cultivars Wanmai29, Zhongyu5, and Shan354 with low POD activity, respectively) at 7, 14, 21, 28, and 35 DAF. The first strand of cDNA was reverse transcribed according to the instructions for the EasyScript One‐Step gDNA Removal and cDNA Synthesis SuperMix. Actin was used as internal reference gene (primer sequences: F: CTATCCTTCGTTTGGACCTTGC; R: AGCGAGCTTCTCCTTTATGTCTC). The qRT‐PCR amplification was performed using TransStart Green qPCR SuperMix, with 20 µL reaction mixture comprising of 2 µL cDNA, 0.4 µL primer (F: TGGAAGGACAAGTTCCTCGC; R: CAGCCCTTGGCGACCTTATC), 10 µL 2×*TransStart* Top/Tip Green qPCR SuperMix, 0.4 µL Passive Reference Dye (50×), and nuclease‐free water to fill to the final total volume. The qPCR reaction followed a two‐step method: pre‐denaturation at 94°C for 30 s, followed by 40 cycles of 94°C for 5 s and 60°C for 30 s. The internal reference gene for fluorescence quantification of wheat gene was actin, and three replicates were set up in the experiment. The expression level of the gene was analyzed by employing the 2^−ΔΔCt^ method.

### 2.7 Statistical analysis

An analysis of variance (ANOVA) was carried out using IBM SPASS Statistics27 software. 228 wheat cultivars and advanced lines were used to verify the association between POD activities.

## 3 RESULTS

### 3.1 Characterization of DNA sequences of *TaPod‐A3*

The full length of the gDNA sequence of the *TaPod‐A3* gene (TraesCS7A02G339600) was amplified. Primer sets A1, A2, and A3 were used to produce PCR fragments of 1595, 1049, 646 bp, respectively. The PCR fragments amplified by the primary of A1 and A2 had 190 bp overlaps, while the primary of A2 and A3 had 133 bp overlaps (Table 1). *TaPod‐A3* was found to encode a protein with 357 amino acids, molecular weight of 30.96 kDa, and p*I* index of 6.82. *TaPod‐A3* amino acids (AA) was predicted to be an unstable protein by https://web.expasy.org/cgi‐bin/protparam/protparam. Meanwhile, Cell‐PLoc 2.0 (http://www.csbio.sjtu.edu.cn/bioinf/Cell‐PLoc‐2/) was used to predict the subcellular localization of the proteins encoded by the *TaPod‐A3* gene. The results showed that the protein encoded by the gene played a role in cytoplasm. The *TaPod‐A3* gene has two exons and one intron (Figure S1), and in the same evolutionary branch as the corresponding gene in Urartu wheat (TuG1812G0700003694.01), it has been hypothesized that there is a close relationship between the subject and (*Triticum urartu*, AA, 2*x* = 14), which is the donor of the A genome for tetraploid wheat (AABB) and hexaploid wheat (AABBDD). This relationship is supported by the findings of Ling et al. (2018), who concluded that there is an evolutionary relationship between the two (Figure S2).

**TABLE 1:** The sequences of the primer sets used in the cloning of the *TaPod‐A3* gene along with their polymerase chain reaction (PCR) profiles and product sizes.

Primer set	DNA sequence of primer (5′‐3′)	Amplified region	Size of PCR	PCR annealing
				Temperature(°C)
A1	Forward: ACTCATTATTGCAGTGCAGTTG	1–1596	1596	56°C
	Reverse: CTAGTGTGACGCTGCTGTTTAA			
A2	Forward: ATGCACAGATGAATGCTACGTCG	1406–2454	1049	57.5°C
	Reverse: TTGTCCTTCCACAGCGTCTCGTT			
A3	Forward: CTGGACAACAACTACTACAAGC	2321–2966	646	53°C
	Reverse: CTTCATAATATGAAATTTTATTAA			

The gDNA sequence of *TaPod‐A3* was comprised of 2967 bp, with two exons and one intron. The intron sequences bordering the exon–intron junctions conformed to the GT‐AG rule. The cDNA sequence was 1496 bp long, while the coding sequence (CDS) region was 1061 bp (Figure S3). The *TaPod‐A3* gene had one single nucleotide polymorphism (SNP) and eight indel, with the indels causing changes in a large number of amino acids (Figures S4 and S5).

### 3.2 Allelic combination of POD genes *TaPod‐A1*, *TaPod‐A3*, and *TaPod‐D1* functional marker in wheat cultivars and advanced lines

**FIGURE 1:**
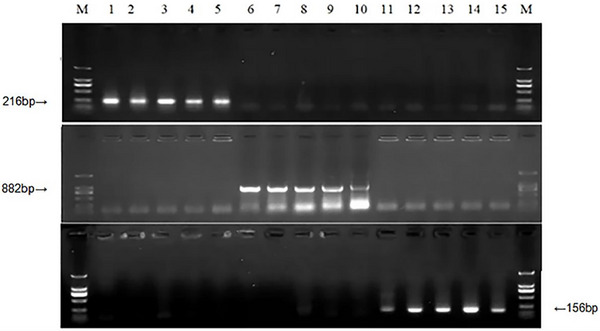
Polymorphisms of polymerase chain reaction fragments amplified by *POD‐7A1* (a), *POD‐7A2* (b), and *POD‐7A3* (c) in five cultivars with lower peroxidase (POD) activities, middle POD activities, and six with higher POD activities, respectively. M: DNA ladder DL2000; 1: Bima1; 2: CA0998; 3: Fu936; 4: Hupei5; 5: Jing9428; 6: Aifeng3; 7: Beijing0045; 8: Chuanmai41; 9: Emai21; 10: Lumai9; 11: Su0663; 12: Tianshan1; 13: Wennong14; 14: Xinmai19; 15: Xinnong291.

Based on polymorphisms between the various sequences of the *TaPod‐A3* gene, we designed and applied the primers *POD‐7A1*, *POD‐7A2*, and *POD‐7A3*. *POD‐7A1* (Table 2), designed from the *TaPod‐A3a* sequence, amplifies a 216‐bp PCR fragment in wheat cultivars and advanced lines with low POD activity, while yielding no PCR product in the wheat cultivars and advanced lines with middle and high POD activity. In contrast, *TaPod‐A3b* amplifies 882‐bp PCR fragment in wheat cultivars and advanced lines with middle POD activity, and *TaPod‐A3c* amplifies a 156‐bp PCR fragment with high POD activity (Figure 1). A total of 228 wheat cultivars and advanced lines were screened using the POD gene markers *POD‐7A1*, *POD‐7A2*, and *POD‐7A3*. Amplification products of 216, 882, and 156 bp were detected in 113 (49.5%), 17 (7.5%), and 98 (43.0%) of the lines, respectively (Tables 2 and 3). The ANOVA showed that the average POD activity for *TaPod‐A3a* (668.6 U min^−1^ g^−1^) was significantly lower than for *TaPod‐A3c* (731.2 U min^−1^ g^−1^) (*p* < 0.05). Therefore, the results show that *TaPod‐A3c* is a favorable allele for POD activity in wheat cultivars and advanced lines, and the gene‐specific functional markers *POD‐7A1*, *POD‐7A2*, and *POD‐7A3* can be used for wheat‐assisted breeding selection.

**TABLE 2:** Molecular markers and primer sequences used to detect peroxidase (POD) active genes.

Marker name	Primer sequence(5′‐3′)	Product size(bp)	Allele	Annealing temperature
POD‐7A1	F: CACGAGACGCTGTGGAAGGACAG	216 bp	*TaPod‐A3a*	68°C
	R: TCGCATTCAAGGACGCATACA			
POD‐7A2	F: TATTTTTTTTTTTTTTGCGTTC	882 bp	*TaPod‐A3b*	66°C
	R: GGATCTCCCCCTTGCGTGCCGGTCTT			
POD‐7A3	F: AAGACCGGCACGCAGGGGGAGA	156 bp	*TaPod‐A3c*	65°C
	R: TCGCATTCAAGGACGCATACA			
POD‐3A1	F: ACGGGAGACGACGAGAAGCAAAGA	291 bp	*TaPod‐A1a*	68°C
	R: TCGTGGAAGTGTAGGCGAAGA			
POD‐3A2	F: GTGGCGCAGGGCCTGTCA	766 bp	*TaPod‐A1b*	62°C
	R: GTTGTCGAACACGTTGGGGGA			
POD‐7D1	F: GCTTCGTCCAGGACGCGTT	540 bp	*TaPod‐D1a*	62°C
	R: CGAGGAATGGGGGGTTGATG			
POD‐7D6	F: TGGGCATGGGGCTTCTGCA	640 bp	*TaPod‐D1b*	58°C
	R: GCGAGGAATGGGGGGTTGATG			

**TABLE 3:** Peroxidase (POD) activity and distribution frequency of different types of Chinese winter wheat cultivars and advanced lines.

Allelic variation	Total			
	Number	Frequency (%)	Mean POD activity	Range
*TaPod‐A1a*	116	50.9	663.4abc	413.3–1009.3
*TaPod‐A1b*	112	49.1	730.4bcde	516.6–1111.8
*TaPod‐A3a*	113	49.5	668.6abcd	431.3–909.3
*TaPod‐A3b*	17	7.5	679.7abcde	598.8–841.8
*TaPod‐A3c*	98	43.0	731.2bcde	461.4–1111.8
*TaPod‐D1a*	138	60.5	678.5abcde	431.3–981.8
*TaPod‐D1b*	90	39.5	723.7abcde	517.1–1111.8
*TaPod‐A1a/TaPod‐A3a/TaPod‐D1a*	38	16.7	625.7a	431.3–787.0
*TaPod‐A1a/TaPod‐A3a/TaPod‐D1b*	16	7.0	645.1abc	517.1–865.4
*TaPod‐A1a/TaPod‐A3c/TaPod‐D1a*	33	14.5	677.7abcde	461.4–939.7
*TaPod‐A1a/TaPod‐A3c/TaPod‐D1b*	21	9.2	721.7abcde	581.1–1009.3
*TaPod‐A1a/TaPod‐A3b/TaPod‐D1b*	5	2.2	690.0abcde	634.7–809.6
*TaPod‐A1a/TaPod‐A3b/TaPod‐D1a*	3	1.3	627.1ab	598.8–676.3
*TaPod‐A1b/TaPod‐A3b/TaPod‐D1a*	7	3.1	702.3abcde	615.5–841.8
*TaPod‐A1b/TaPod‐A3b/TaPod‐D1b*	2	0.9	653.6abc	612.9–694.3
*TaPod‐A1b/TaPod‐A3a/TaPod‐D1a*	35	15.4	677.8abcde	516.6–862.2
*TaPod‐A1b/TaPod‐A3a/TaPod‐D1b*	24	10.5	738.5cde	534.9–909.3
*TaPod‐A1b/TaPod‐A3c/TaPod‐D1a*	22	9.6	771.2de	571.5–981.8
*TaPod‐A1b/TaPod‐A3c/TaPod‐D1b*	22	9.6	780.6e	582.3–1111.78

*Note*: Different letters mean the difference is significant a/b, lowercase letters reflect a *p *< 5% significant level.

Among the 228 wheat cultivars and advanced lines, the *TaPod‐A1b/TaPod‐A3c/TaPod‐D1b* allele combination had significantly (*p* < 0.05) higher POD activity (780.6 U min^−1^ g^−1^ on average) than the combination *TaPod‐A1a/TaPod‐A3a/TaPod‐D1a* (625.7 U min^−1^ g^−1^ on average). The POD activities of the allele combinations *TaPod‐A1a/TaPod‐D1a*, *TaPod‐A1a/TaPod‐A3a*, and *TaPod‐A3a/TaPod‐D1a* were lower than those of *TaPod‐A3c/TaPod‐D1b*, *TaPod‐A1b/TaPod‐A3c*, and *TaPod‐A1b/TaPod‐D1b*. In addition, the POD activity with two favorable allele combinations was significantly higher than that with two non‐favorable allele combinations, and the POD activity of *TaPod‐A1b/TaPod‐A3c* (775.9 U min^−1^ g^−1^ on average), *TaPod‐A1b/TaPod‐D1b* (754.2 U min^−1^ g^−1^ on average), and *TaPod‐A3c/TaPod‐D1b* (751.8 U min^−1^ g^−1^ on average) was significantly higher than that of *TaPod‐A3a/TaPod‐D1a* (650.7 U min^−1^ g^−1^ on average), *TaPod‐A1a/TaPod‐D1a* (649.0 U min^−1^ g^−1^ on average), and *TaPod‐A1a/TaPod‐A3a* (631.5 U min^−1^ g^−1^ on average) (Figure S6). The frequency distribution of wheat cultivars and advanced lines with *TaPod‐A1a/TaPod‐A3a/TaPod‐D1a* was the highest (38, 16.7%), whereas that of *TaPod‐A1b/TaPod‐A3b/TaPod‐D1b* was the lowest (2, 0.9%). It was concluded that the use of an excellent allele combination with high POD activity could significantly increase the activity of POD (Figure S6; Table 3).

### 3.3 Expression analysis of *TaPod‐A3* gene in different wheat tissues during the flowering stage

**FIGURE 2:**
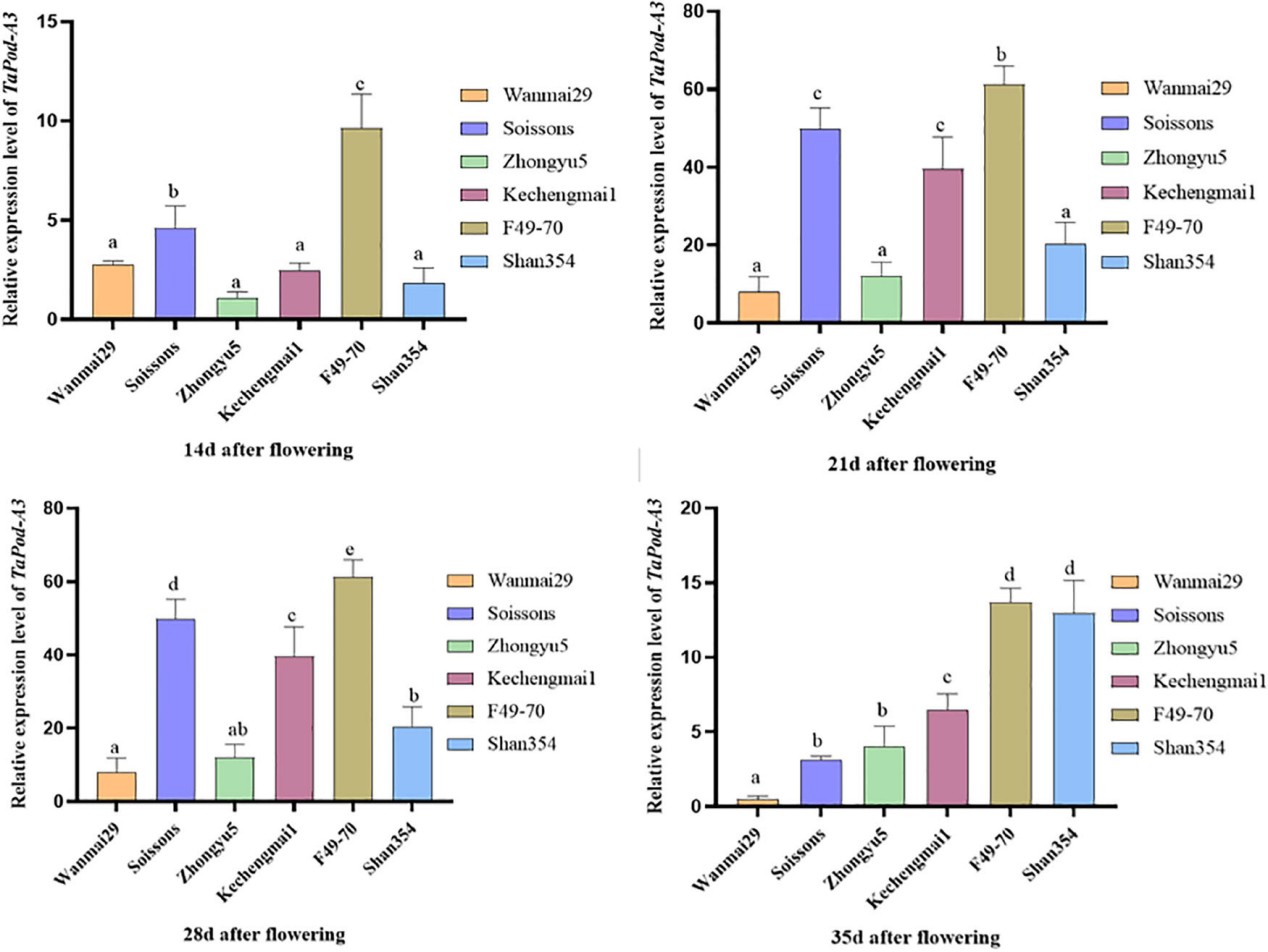
Correlation expression levels of the *TaPod‐A3* gene at different stages. Different letters represent significant differences.

In order to investigate the expression patterns of *TaPod‐A3* genes during grain development, six wheat cultivars and advanced lines with extreme POD activity were analyzed at five time points of 7 (control), 14, 21, 28, and 35 DAF. Gene expression levels were quantitatively analyzed at each stage and then compared with the levels observed at 7 DAF. (Figure 2). The expression levels of the beneficial allele with high POD activities in wheat cultivars and advanced lines (Soissons, Kechengmai1, and F49‐70) were higher than that of the nonoptimal allele with low POD activity (Wanmai 29, Zhongyu 5, and Shan 354) in all periods. In particular, the highest expression level of wheat cultivar Sossions was about 25 times that of the lowest expression of wheat cultivar Wanmai 29 at 21 DAF. These results showed that there was a positive correlation between genotype and expression levels.

**FIGURE 3:**
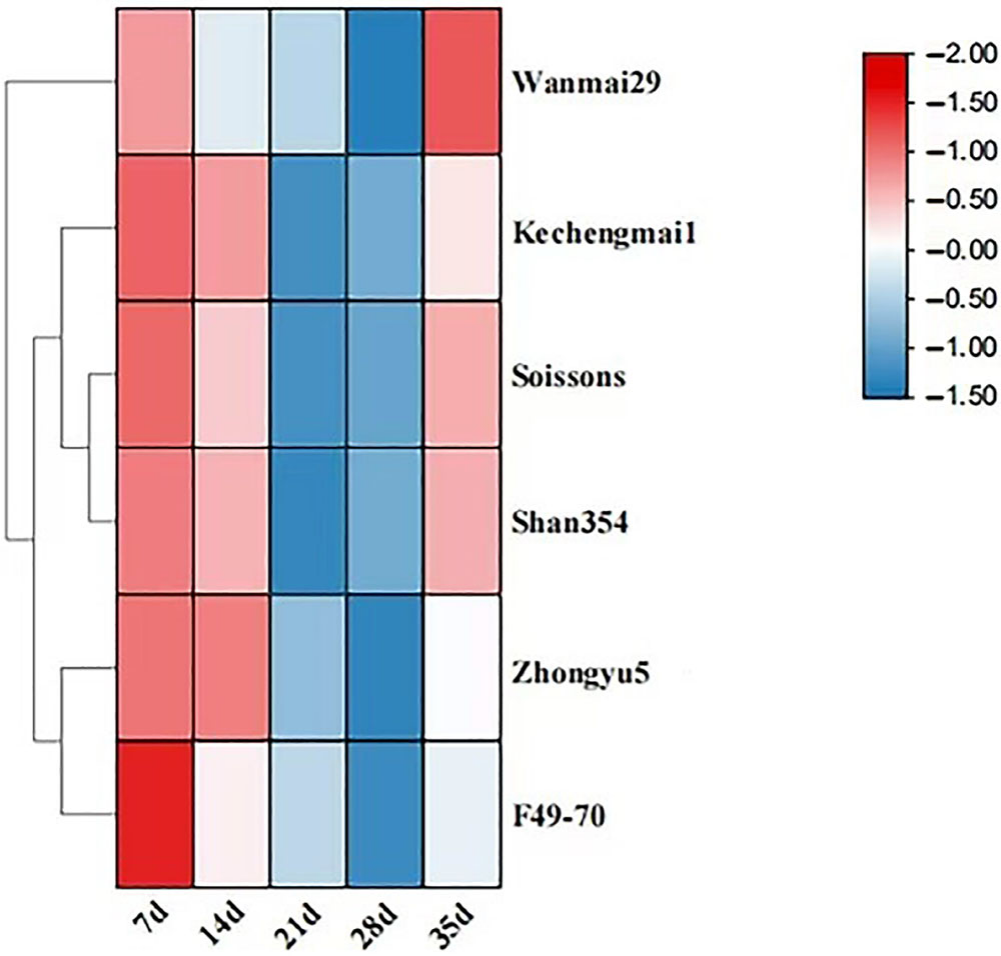
Heatmap of *TaPod‐A3* gene expression analysis.

Overall, the POD genes are up‐regulated at 7, 14, and 21 days, down‐regulated gradually at 28 and 35 days, and were the highest at 21 DAF. The *TaPod‐A3* expression of the Sossions wheat cultivar was the highest among the six wheat cultivars and advanced lines at 21 DAF. At 21 and 28 DAF, these results indicated that this gene may be involved in catalyzing the synthesis of wheat grains, and its expression also has certain tissue specificity. We have visualized the expression patterns of POD genes at different stages of flowering as a heatmap (Figure 3).

## 4 DISCUSSION

### 4.1 Implication of functional markers for POD activity in wheat breeding

Class III POD in wheat grains not only participates in the seed development process but also affects the processing quality of flour (Borrelli et al., 2003; Takasaki et al., 2005). In view of the dual effects of oxidation and reduction of POD on wheat quality (Zhou et al., 2021), it is of great practical significance to explore the characteristics of POD activity‐related genes for wheat quality improvement by molecular marker‐assisted breeding.

The present study bridges a critical gap between POD gene polymorphism and flour quality by establishing the first functional markers for *TaPod‐A3* allele selection, revealing epistatic interactions among POD loci, and linking expression patterns to phenotypic outcomes. The development of functional markers (POD‐7A1/2/3) enabled the effective differentiation of wheat lines exhibiting differential POD activities. The *TaPod‐A3c* genotype demonstrated higher activity than the *TaPod‐A3a* genotype (731.2 > 668.6 U·min^−1^·g^−1^). This finding serves to validate *TaPod‐A3* as a genetic hotspot for quality improvement. It was found that a significant number of insertions and deletions (indels) occurred in the *TaPod‐A3* gene, resulting in multiple amino acid changes. This may be an important reason why the POD activity of wheat cultivars and advanced lines with the allele *TaPod‐A3c* was higher than that of wheat cultivars and advanced lines with the allele *TaPod‐A3a*. Indel is a highly severe mutation that causes a frame shift and a misalignment of the order of all bases after insertion or deletion of a site. This in turn causes a large number of amino acids to alter the properties of proteins (Khadka et al., 2017). SNPs and Indels represent the most prevalent genetic markers employed in the context of breeding selection (Fan et al., 2019). A high‐density genetic linkage map was constructed using 5081 SNPs from genotyping by sequencing. Haplotype analysis indicated that the QTL combinations significantly improved yield and grain traits (Z. Wang et al., 2022). Research has demonstrated that the absence of two amino acids in MtrA impacts its physiological function, resulting in phenotypic alterations (Pan et al., 2019).

### 4.2 Allelic combination of POD genes *TaPod‐A1*, *TaPod‐A3*, and *TaPod‐D1* functional marker in wheat cultivars and advanced lines

Flour color is a quantitative trait and is influenced by genetic and environmental factors, including genotype (variety), climate (light, temperature, precipitation, etc.) and cultivation conditions (planting year, location, water, fertilizer, cultivation measures, etc.) (Deng et al., 2016). In this study, the average POD activity of wheat cultivars and advanced lines with multiple favorable alleles was found to be significantly higher (*p* < 0.05) than that of wheat cultivars and advanced lines with multiple non‐favorable alleles (780.6 U min^−1^ g^−1^ < 625.7 U min^−1^ g^−1^), indicating that gene polymerization increased the activity of POD to a certain extent. The allele stacking (*TaPod*‐*A1b/A3c/D1b*) provides a molecular blueprint for breeding high‐quality wheat. Breeding wheat cultivars and advanced lines and lines using haplotype‐specific marker combinations in rice has also confirmed that the 1000‐grain weight of these lines is much higher than that of the other kinds of lines (G. Liu et al., 2023). The combination of favorable alleles for POD activity in wheat cultivars and advanced lines can significantly improve the POD activity of wheat cultivars and advanced lines and can make up for the lack of unit point selection, thereby improving the effectiveness of screening germplasm with high POD activity (Altın et al., 2017; Bagge et al., 2007). Therefore, we should aggregate wheat cultivars and advanced lines with high POD activity to strengthen the improvement of wheat flour quality.

### 4.3 Characteristics of the *TaPod‐A3* gene expression at different wheat cultivars and advanced lines and periods after flowering

Due to its simplicity, rapidity, reliability, and high sensitivity, qPCR has been widely adopted as the gold‐standard method for species‐specific detection (Han et al., 2023; McCarthy et al., 2023). Through the analysis of expression patterns of POD genes, it was found that the expression levels of different genotypes were different in different periods after flowering, but the expression level of the *TaPod‐A3* gene was expressed in wheat for five stages after flowering, reflecting specific spatiotemporal expression characteristics. Among them, the expression levels at 21 and 28 days periods were about six to eight times those in the 14 and 35 days periods. Meanwhile, we found that the endosperm of wheat grains at this stage became gluten‐like, and the increase of POD activity was one of the factors that changed the rheological properties of wheat with the increase of gluten content, which was consistent with the characteristics of wheat grain development and growth in the grain filling stage (Gao et al., 2020; Li et al., 2020; Y. Wang et al., 2015). Transcriptome analysis of F‐box genes in wheat at different developmental stages showed that some F‐box genes were differentially expressed in one or more developmental stages of wheat, which may be due to the great changes in the cell composition of seeds at maturity or grain filling stages (Hong et al., 2020). At 21 and 28 DAF, the expression levels of wheat cultivars and advanced lines with high POD activity, namely, Soissons, kechengmai1, and F49‐70 (*TaPod‐A3c*), were significantly higher than those of wheat cultivars and advanced lines with low POD activity (Wanmai 29, Zhongyou 5, and Shan 354) (*TaPod‐A3a*) (*p* < 0.05), indicating that the relative expression levels of different alleles were consistent with the phenotypic characteristics of POD activity in the previous period, suggesting that the *TaPod‐A3* gene may be involved in catalyzing the cross‐linking reaction of POD activity in wheat grains. The results of this study are of great significance for improving wheat germplasm resources and breeding high‐quality wheat cultivars and advanced lines. *TaPod‐A3* expression profiling offers a biomarker for early selection in breeding programs. The expression degree and expression pattern of different genes are different, and by clarifying the expression patterns of these genes, it further provides a basis for functional research and promotes their potential applications in wheat genetic improvement. While the present study confirmed a significant correlation between the *TaPod‐A3* genotype and POD activity through correlation analysis, transgenic validation remains an important direction for future research.

## 5 CONCLUSION

In this study, the full‐length sequence of *TaPod‐A3* gene was cloned, and bioinformatics analysis was carried out to clarify its structural distribution and facilitate the next functional elucidation. The functional markers *POD‐7A1*, *POD‐7A2*, and *POD‐7A3* of *TaPod‐A3* gene were developed and verified by 228 wheat cultivars and advanced lines, and the average activity of wheat cultivars and advanced lines with *TaPod‐A3b* and *TaPod‐A3c* alleles was higher than wheat cultivars and advanced lines with low *TaPod‐A3a* allele. The results indicated that the marker could be effectively applied to molecular marker‐assisted breeding of wheat's fast breeding process.

## AUTHOR CONTRIBUTIONS

**Xinyuan Liu**: Conceptualization; data curation; formal analysis; writing—original draft. **Zhaoqiang Wang**: Investigation; supervision; validation. **Lili Wang**: Data curation; methodology; validation; visualization. **Yukun Cheng**: Investigation; visualization. **Bin Bai**: Validation; visualization; writing—review and editing. **Hongwei Geng**: Resources; supervision; writing—review and editing. **Mengyao Ma**: Conceptualization; formal analysis.

## CONFLICT OF INTEREST STATEMENT

The authors declare no conflicts of interest.

## Supporting information




**Supplementary Figure S1**. Schematic diagram of intron‐exon structure between wheat species.

**Supplementary Figure S2**. Schematic diagram of the phylogenetic tree among wheat species.

**Supplementary Figure S3**. The gDNA and cDNA sequence structure of the *TaPod‐A3* gene.

**Supplementary Figure S4**. Alignment of the alleles *TaPod‐A3a* and *TaPod‐A3b* and *TaPod‐A3c*.

**Supplementary Figure S5**. Comparison of Amino acid sequences *TaPod‐A3a* and *TaPod‐A3b* and *TaPod‐A3c*.

**Supplementary Figure S6**. Average POD activities of wheat varieties with different allele combinations.

Supplementary Material

## Data Availability

The datasets generated or analyzed during this study are included in this article and its Additional file or are available from the corresponding author on reasonable request.
