# Supplementary material for: Development of functional markers and expression analysis for a Peroxidase gene TaPod‐A3 on chromosome 7AL in wheat
Source: Plant Genome. 2025 Aug 30;18(3):e70103. doi: 10.1002/tpg2.70103 (PMC12397898; doi:10.1002/tpg2.70103)
Supplement: Supplementary file 2 [file TPG2-18-e70103-s004.docx]

>7A dna: chromosome chromosome: IWGSC:7A: 497776003:497778969:1(green indicates exons and yellow indicates intron regions).

ACTCATTATTGCAGTGCAGTTGACGCTCACTGTGCCGGGCTTGCGAGGGTGAGCGATACC

AGCTAGCCACAGCGCACGTACGACGGCCATGGCGAGCAGCGGCGGGCTGGCCGGTCTGGC

GGTGCTGCTCACCGCCGTGCTGTGCCTGCAGCTGCCGGTCCACAGCCGCGCGCAGCTGCG

CGTGGGGTTCTACAACACCACCTGCCCCAACGCCGAGGCCATCGTCCGGCAGGCCGTCAC

GGCCGCCTTCGCCACCAACCCCGGCGTCGCCGCCGGCCTCATCCGCCTCCACTTCCACGA

CTGCTTCGTCGAGGTACGTGCGTGCGTACCTGTTGCATGTACAGGATCAGGAGCAGTTGC

ATCGTAGTTGTTTTGCTATGTTTTTTTCTTAAAAATAGACGCTCATAACGTGCATGCATA

CGCATCTTTTTTTCACGTCCAGATTTGGTTGCTGAATTTTTTTTTGAAAGTAATTTGGTT

GCTGAAATTAAATAGTTCATGTAGTTCCCAAACTCCTTATCCATCTTCCTAACTTTATTG

TTGGCCTGAAACTGAAATGGAGAAGAAACTAAACAGGAGTCTCTGGAGAAGATAGTGTGG

CAACTAGAAACTTTCCACATAACTAATAATAACTTTACATCTGCGTCAGGGTGTTAGCGA

CAAGCTTGTAATCCTTCGTGAAAGGGAAAGCCGTGCTAAAAGGTGTGCACAAAGACAATT

TACTTGAACATAACGATTTTGAGTTGAACAACACGGTGGGCGACGATGACTACTAACCTA

ATCAATTGATCGAGTGAACATAGTGGAGCTCACTCTTGTTTTGACCACATATCGTACGTT

GACGACGTGTCACCTCGCTAGTTTGGTAGGTCGGGTGTCATGCTTCACATGTTTGGACTA

TTCTCGTGGGTTAGACAACTATACATGTGACTACTAAATATCTACGAATATCAGTACTAC

AGATGATTAGACGTATATCAGTACTCCCTCCATTTTCTTTTACTGTGCGTATGAGTTTTG

TCTGAAATCAAGCTTTGTAGAGTTTGATCAACTTTATAGAAAAAATATCTACATTCACAA

TACAAAATCAATATGATTAGATGCATGCAATTATTTTTTATCCTATATAACTTTAGTATT

ATACATATGTTGGTATTTTTTTAATATAAATTCGATCAAACTTTATGAAGTTTGATTTTG

TATAAAATCATATGAGAAGTAAAAAGAAACGAATTGAGACATACTACTTGCTAGGTCGTA

CCTAGCTAGCATCTGGTAGGTTAGGTGTCACATTTTCACATATATGGACTATTCAGTGAG

TTAGACAATTATCCACATACTTGTCTGAAGTATACGTACATGCGACTACCGAATACTCTA

ACTAGGACTAGTCAACATGGTATATGATGCACAGATGAATGCTACGTCGTACTCCGTCTG

CAAGCTACCTACTACTATGTCATGGACGTAGGTGGAAAATGTCTAATTTGTCTCCGTCTG

CAAGCTAGCTAGCTAGGTTCAGGAATGCATGGTACGCCGGCAATTTCCTTTCTACTATAA

GCAACAGATTAATTATTAAACAGCAGCGTCACACTAGCAGGTTGAACACGGTGTAGTAGG

AGTAGTATTGGTTATGACTATTTTTTTTTTTTTGCGTTCGTATTGGTTATGACTAGGACA

CCTATGTATAGTTAACCAGTGCAAAAATAATGCATCAAATAACGTAGTATACATCATCTT

AATTGCGGTGCTCTCCATGTGATATGAACCCACCATGCATGCAGGGCTGTGATTCATCGG

TGCTCCTGTCCGTCAACCCCGGCGGAGGCACGACGGAGCGCGAGGCAGCGCCGAACAACC

CGAGCCTGCGCGGCTTCGCGGTCGTGGACGCCGCCAGGGCGGCATTGGAGCAGAGCTGCC

CGCGCACGGTGTCATGCGCCGACATCCTGGCCTTCGCCGCCCGGGACAGCGTGAACATCA

CCGGCAGCAACGCCTTCTACCAGGTCCCTTCCGGCCGCCGCGACGGGAACCTCTCGACCG

ACACCGGCGCGTTCACCCTCCCGGGGCCGAACCTGACGGCGGACGGCCTCGTCAGGGGGT

TCGCGGACCGGAACCTGACCGCCGAGGACATGGTGGTCCTGTCGGGCTCCCACACCCTGG

GCCGCTCCCACTGCAACTCCTTCATCGTCCGGAACCGGGAGCGGCTGGCGAGCGGCACCA

TCAGCCCGGCGTACCAGGCGCTGCTGGAGGCGCTGTGCCCGGCGAACACGAGCCAGTTCA

CCAACGTGACGACGGAGATCGACCTGAGCACGCCGGTGGTGCTGGACAACAACTACTACA

AGCTGGTGCAGCTCAACCTGGGCCTGCACTTCTCCGACGACCAGCTCATCCGCAACGCCA

CCCTCAAGGCCTTCGTCGACGCCTTCGCCGCCAACGAGACGCTGTGGAAGGACAAGTTCC

TCGCCGCCATGATCAAGATGGGCAACATCAGCCCCAAGACCGGCACGCAGGGGGAGATCC

GCCTCAACTGCAGCCTCGTCAACCCGGCCTCCTCTTCTTCGTCCGCCTACGCTGGGGTGA

TCGAGATGCTCCGCCGACAGGGCTCCGACGATAAGGTCGCCAAGGGCTGATGTATGCGTC

CTTGAATGCGAATGCACGGGCGTGCTGGCCGGTCGGTGTCCGTTCGCACGTATACTAGAC

TTGTGATGAATGATGAATAATAAACGCTGTCGTGCTCTGAGTTGCTGAGTCATGCATGGA

CTAGATCTGGTTGGAGAATGAATGGTCCAACACATCACCGGCGCCATGCGCGCGCGTCGC

AGTGCTCCATTACTACGAAGCACCGATACGGCGACATCGATACGACGATACAGATACAGG

GATACGGGATATGGTAATTTTGAAAAACAGCAATACGGCGATACGGCAGGTATATATAAA

TAATTAATAAAATTTCATATTATGAAG

>TraesCS7A02G339600.1 TraesCS7A02G339600:TraesCS7A02G339600.1 cdna:protein_coding (highlighted in yellow as CDS area)

ACTCATTATTGCAGTGCAGTTGACGCTCACTGTGCCGGGCTTGCGAGGGTGAGCGATACC

AGCTAGCCACAGCGCACGTACGACGGCCATGGCGAGCAGCGGCGGGCTGGCCGGTCTGGC

GGTGCTGCTCACCGCCGTGCTGTGCCTGCAGCTGCCGGTCCACAGCCGCGCGCAGCTGCG

CGTGGGGTTCTACAACACCACCTGCCCCAACGCCGAGGCCATCGTCCGGCAGGCCGTCAC

GGCCGCCTTCGCCACCAACCCCGGCGTCGCCGCCGGCCTCATCCGCCTCCACTTCCACGA

CTGCTTCGTCGAGGGCTGTGATTCATCGGTGCTCCTGTCCGTCAACCCCGGCGGAGGCAC

GACGGAGCGCGAGGCAGCGCCGAACAACCCGAGCCTGCGCGGCTTCGCGGTCGTGGACGC

CGCCAGGGCGGCATTGGAGCAGAGCTGCCCGCGCACGGTGTCATGCGCCGACATCCTGGC

CTTCGCCGCCCGGGACAGCGTGAACATCACCGGCAGCAACGCCTTCTACCAGGTCCCTTC

CGGCCGCCGCGACGGGAACCTCTCGACCGACACCGGCGCGTTCACCCTCCCGGGGCCGAA

CCTGACGGCGGACGGCCTCGTCAGGGGGTTCGCGGACCGGAACCTGACCGCCGAGGACAT

GGTGGTCCTGTCGGGCTCCCACACCCTGGGCCGCTCCCACTGCAACTCCTTCATCGTCCG

GAACCGGGAGCGGCTGGCGAGCGGCACCATCAGCCCGGCGTACCAGGCGCTGCTGGAGGC

GCTGTGCCCGGCGAACACGAGCCAGTTCACCAACGTGACGACGGAGATCGACCTGAGCAC

GCCGGTGGTGCTGGACAACAACTACTACAAGCTGGTGCAGCTCAACCTGGGCCTGCACTT

CTCCGACGACCAGCTCATCCGCAACGCCACCCTCAAGGCCTTCGTCGACGCCTTCGCCGC

CAACGAGACGCTGTGGAAGGACAAGTTCCTCGCCGCCATGATCAAGATGGGCAACATCAG

CCCCAAGACCGGCACGCAGGGGGAGATCCGCCTCAACTGCAGCCTCGTCAACCCGGCCTC

CTCTTCTTCGTCCGCCTACGCTGGGGTGATCGAGATGCTCCGCCGACAGGGCTCCGACGA

TAAGGTCGCCAAGGGCTGATGTATGCGTCCTTGAATGCGAATGCACGGGCGTGCTGGCCG

GTCGGTGTCCGTTCGCACGTATACTAGACTTGTGATGAATGATGAATAATAAACGCTGTC

GTGCTCTGAGTTGCTGAGTCATGCATGGACTAGATCTGGTTGGAGAATGAATGGTCCAAC

ACATCACCGGCGCCATGCGCGCGCGTCGCAGTGCTCCATTACTACGAAGCACCGATACGG

CGACATCGATACGACGATACAGATACAGGGATACGGGATATGGTAATTTTGAAAAACAGC

AATACGGCGATACGGCAGGTATATATAAATAATTAATAAAATTTCATATTATGAAG

**Figure S3.** The gDNA and cDNA sequence structure of the *TaPod-A3* gene.
