# Supplementary figures and images for: Development of functional markers and expression analysis for a Peroxidase gene TaPod‐A3 on chromosome 7AL in wheat
Source: Plant Genome. 2025 Aug 30;18(3):e70103. doi: 10.1002/tpg2.70103 (PMC12397898; doi:10.1002/tpg2.70103)

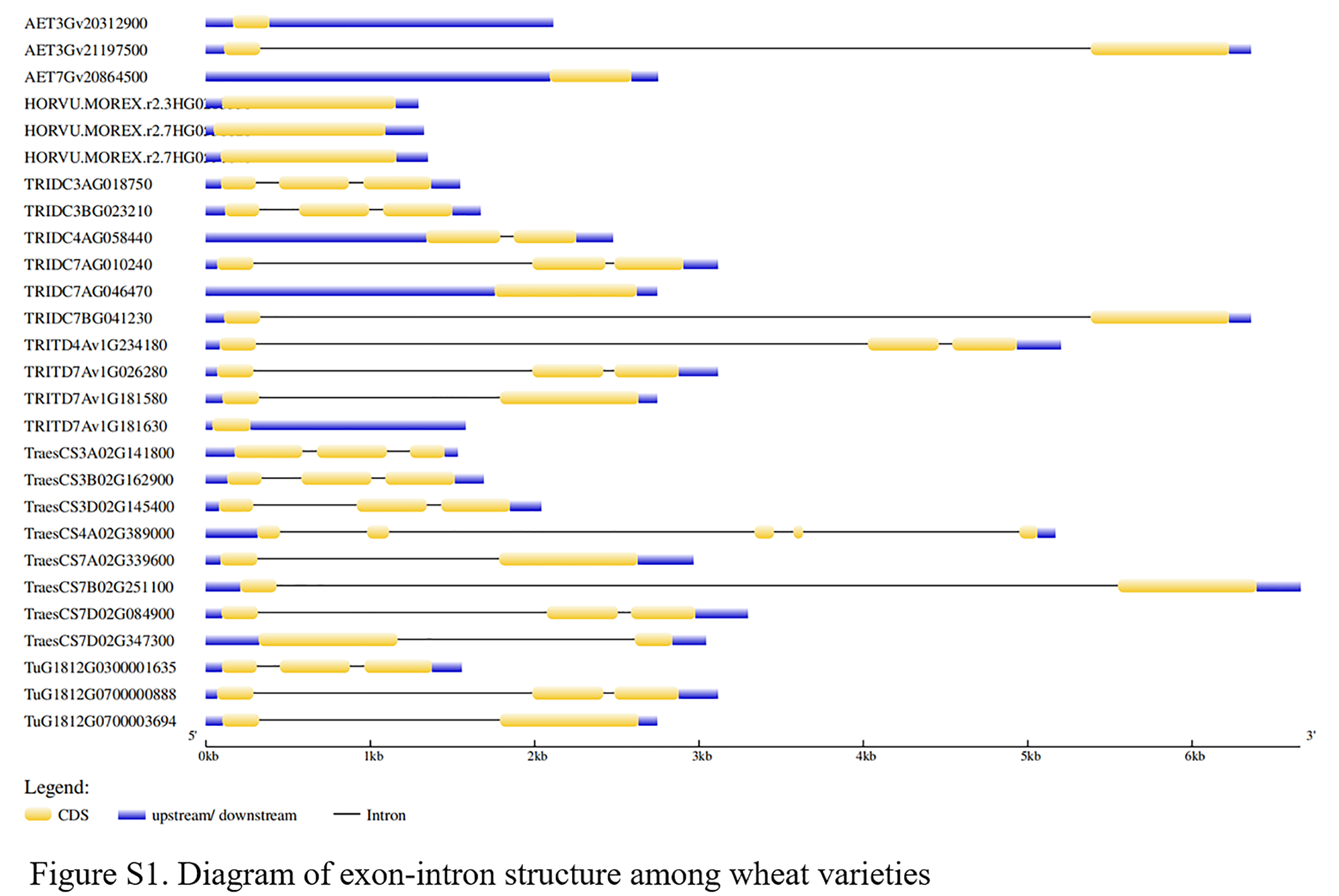

Supplement: Supplementary file 1 — Supplementary Figure S1. Schematic diagram of intron‐exon structure between wheat species. [file TPG2-18-e70103-s002.tif]

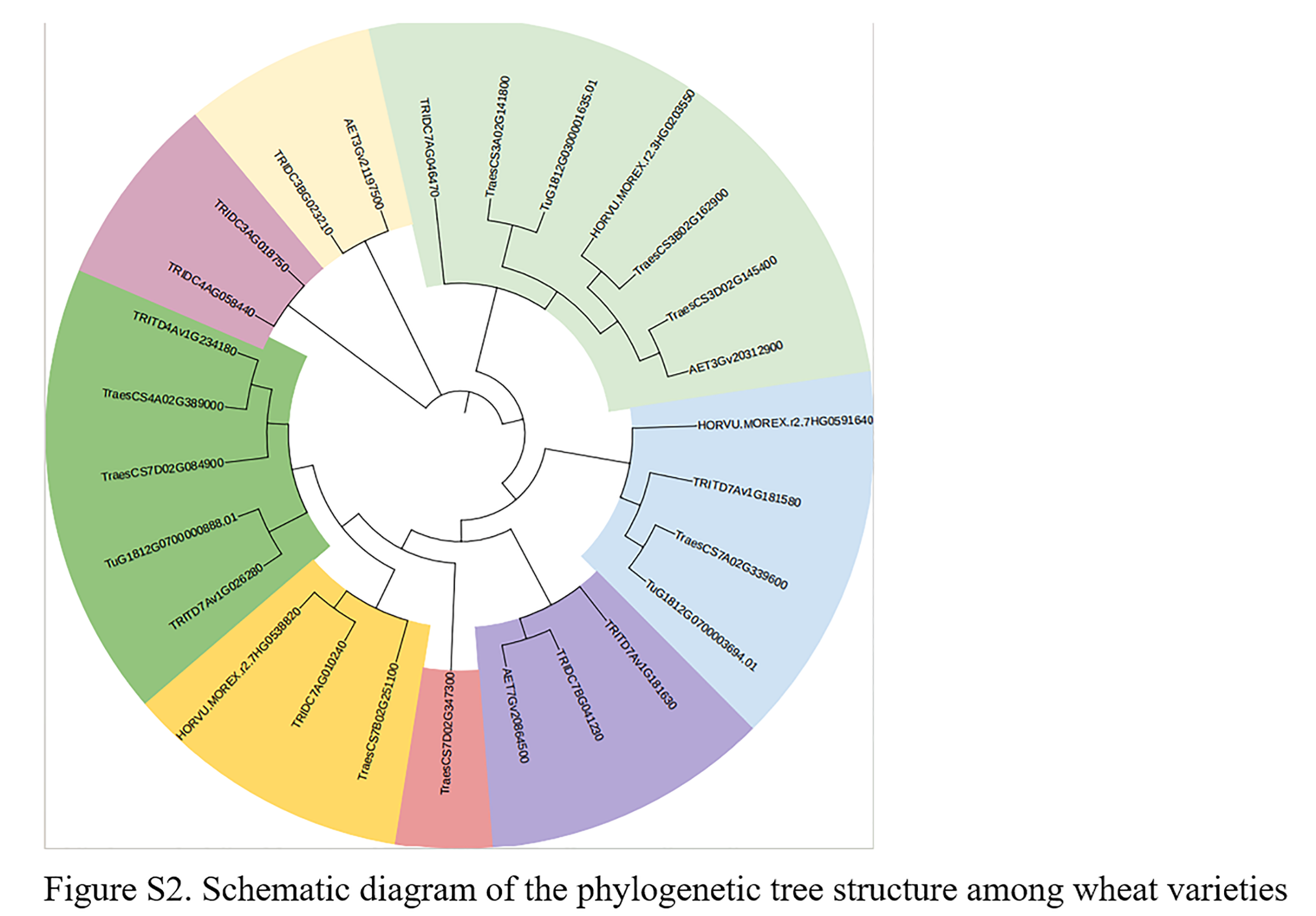

Supplement: Supplementary file 2 — Supplementary Figure S2. Schematic diagram of the phylogenetic tree among wheat species. [file TPG2-18-e70103-s005.tif]

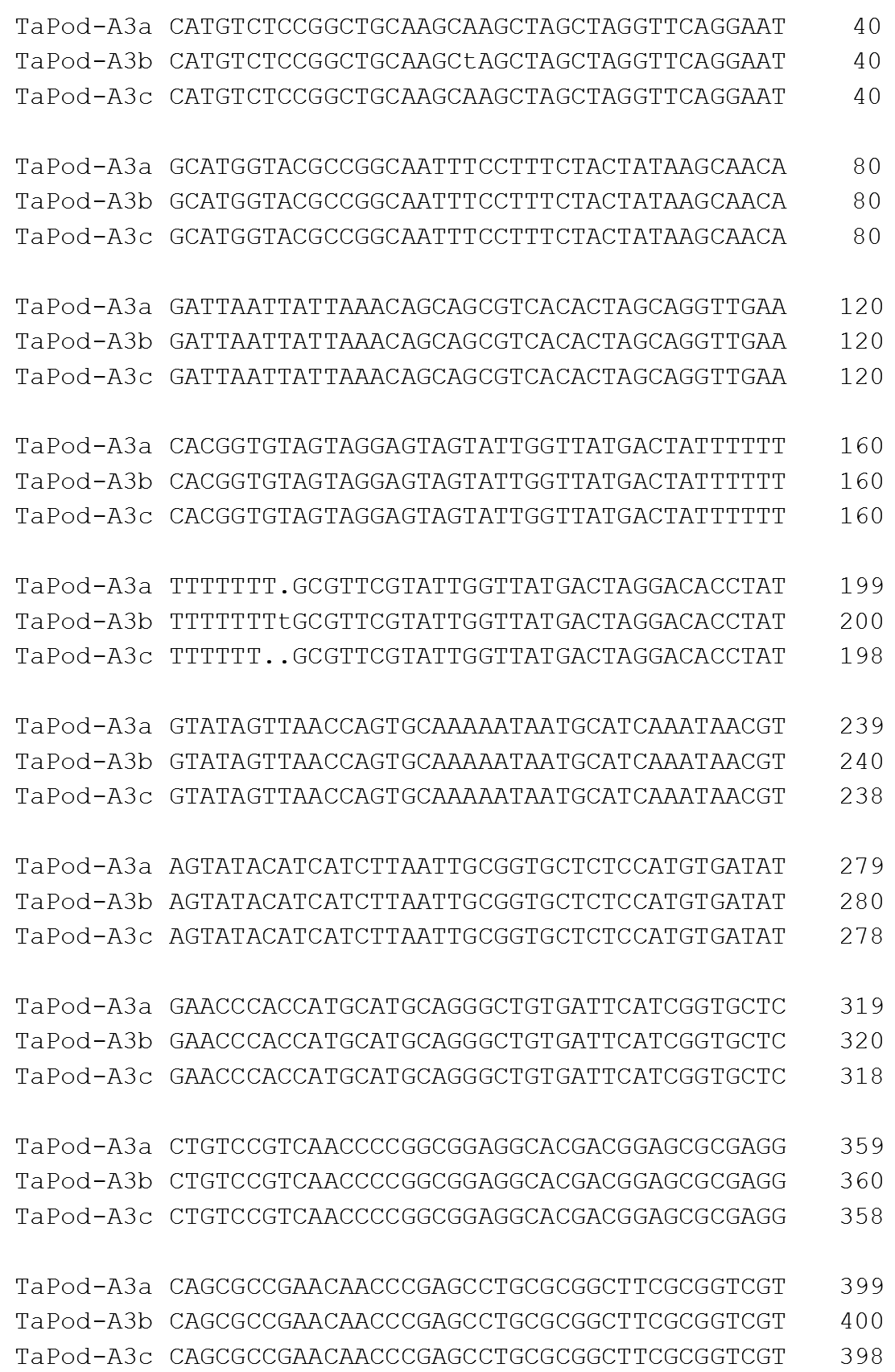


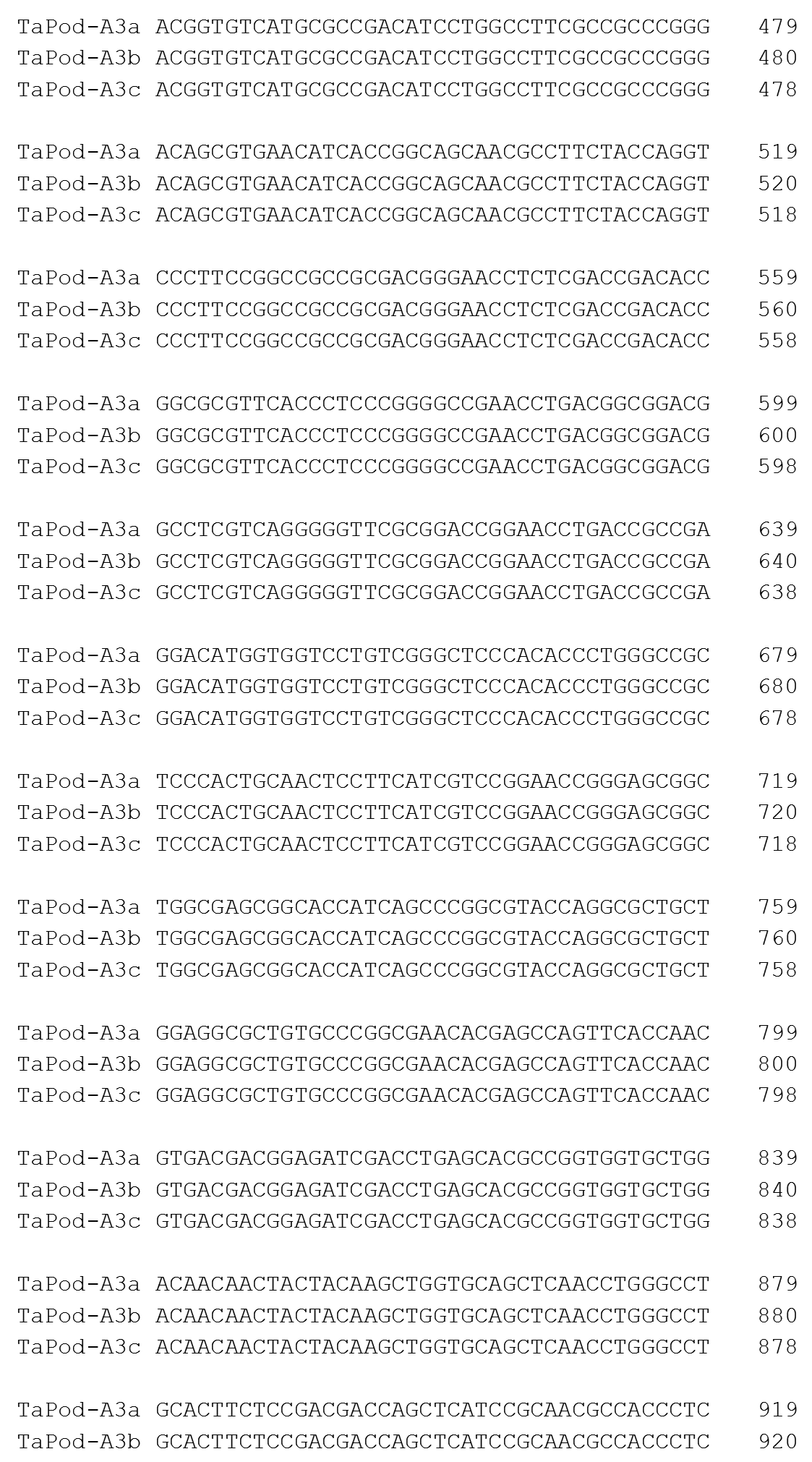


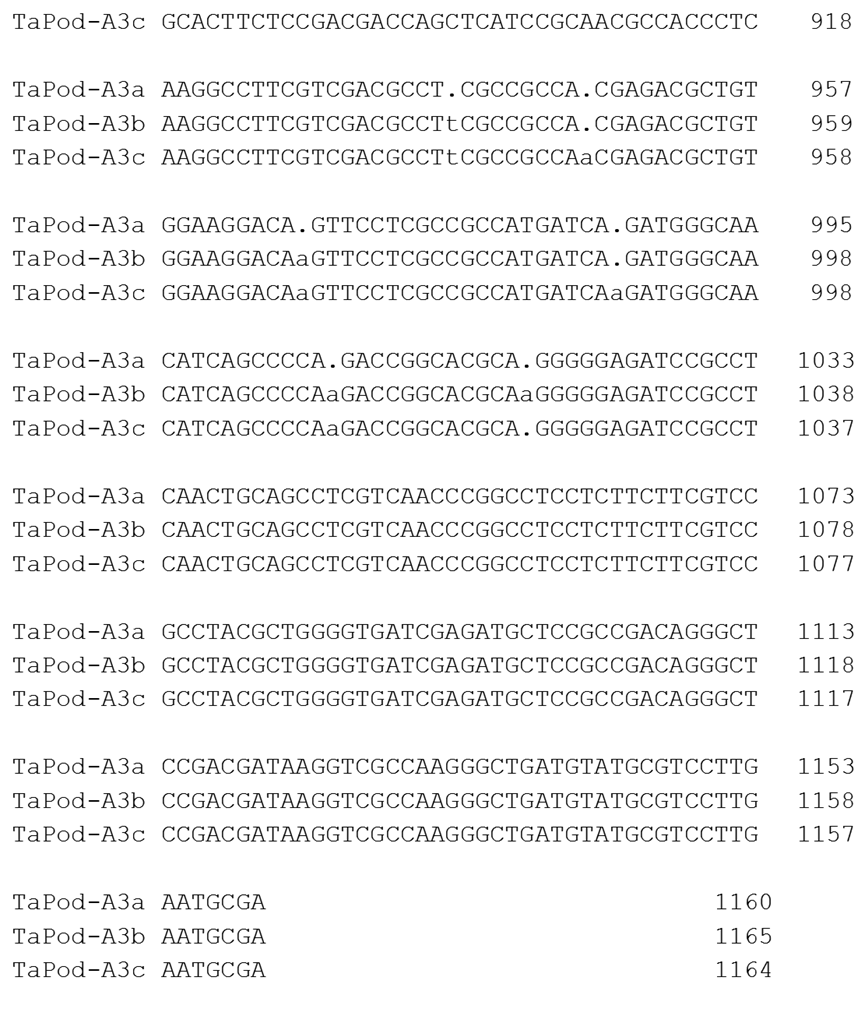


Figure S4 Alignment of the alleles *TaPod-A3a* and *TaPod-A3b* and *TaPod-A3c*

Supplement: Supplementary file 4 — Supplementary Figure S4. Alignment of the alleles TaPod‐A3a and TaPod‐A3b and TaPod‐A3c. [file TPG2-18-e70103-s003.docx]

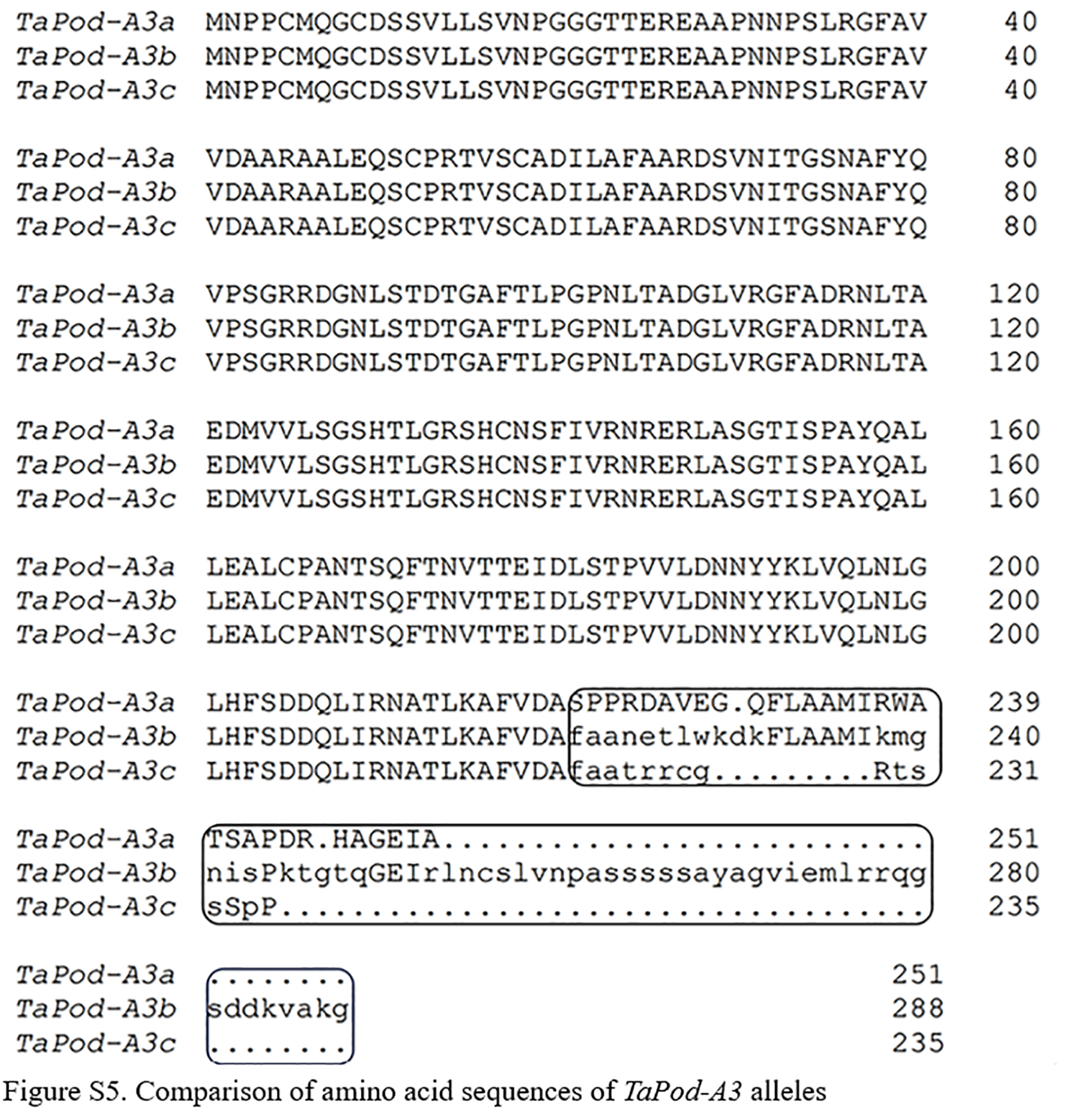

Supplement: Supplementary file 5 — Supplementary Figure S5. Comparison of Amino acid sequences TaPod‐A3a and TaPod‐A3b and TaPod‐A3c. [file TPG2-18-e70103-s007.tif]

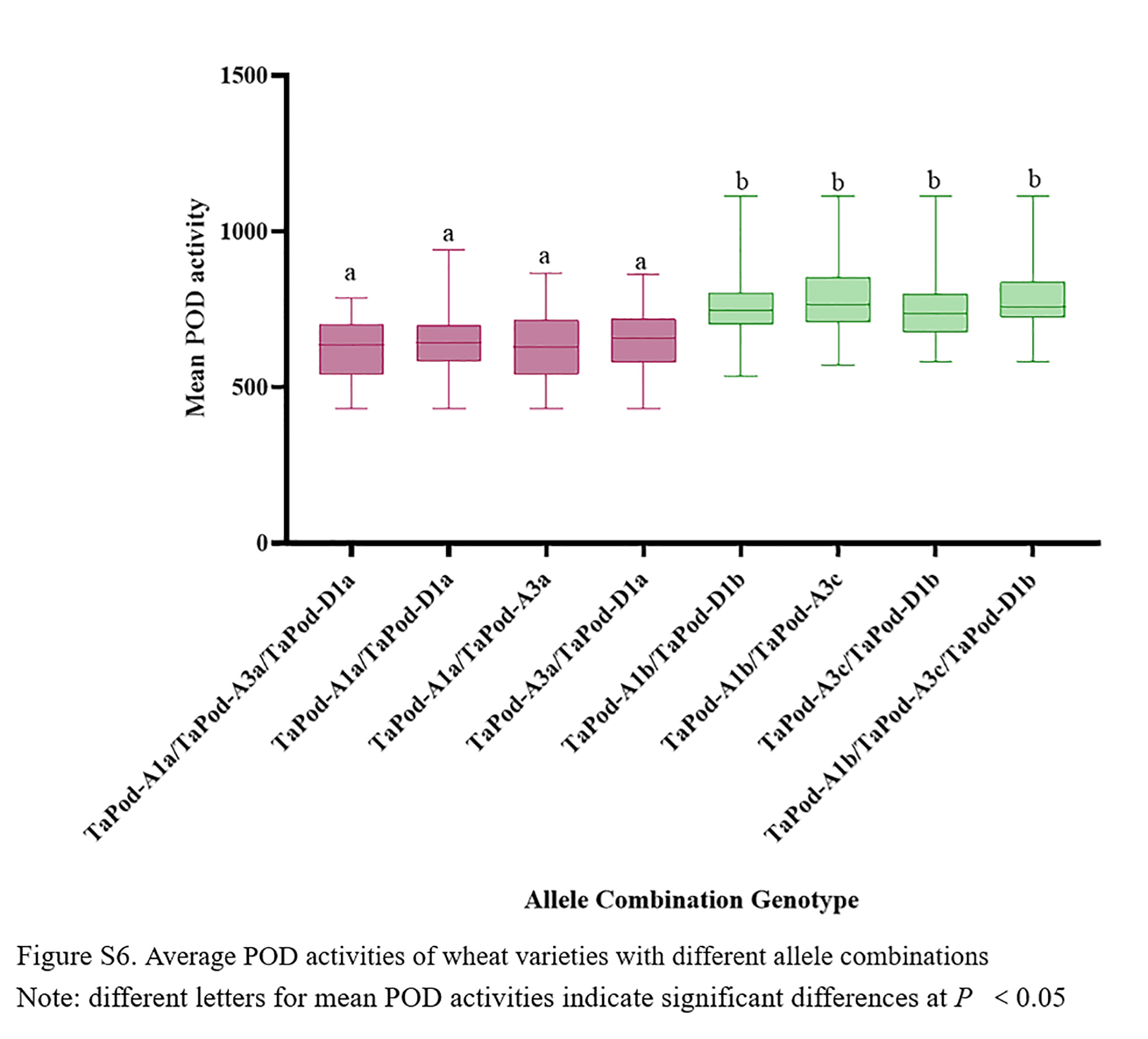

Supplement: Supplementary file 6 — Supplementary Figure S6. Average POD activities of wheat varieties with different allele combinations. [file TPG2-18-e70103-s001.tif]
